# sEMG-Based Gesture Classifier for a Rehabilitation Glove

**DOI:** 10.3389/fnbot.2022.750482

**Published:** 2022-05-30

**Authors:** Dorin Copaci, Janeth Arias, Marcos Gómez-Tomé, Luis Moreno, Dolores Blanco

**Affiliations:** Department of Systems Engineering and Automation, Carlos III University of Madrid, Madrid, Spain

**Keywords:** sEMG, gestures recognition, neural networks, hand rehabilitation, shape memory alloy

## Abstract

Human hand gesture recognition from surface electromyography (sEMG) signals is one of the main paradigms for prosthetic and rehabilitation device control. The accuracy of gesture recognition is correlated with the control mechanism. In this work, a new classifier based on the Bayesian neural network, pattern recognition networks, and layer recurrent network is presented. The online results obtained with this architecture represent a promising solution for hand gesture recognition (98.7% accuracy) in sEMG signal classification. For real time classification performance with rehabilitation devices, a new simple and efficient interface is developed in which users can re-train the classification algorithm with their own sEMG gesture data in a few minutes while enables shape memory alloy-based rehabilitation device connection and control. The position of reference for the rehabilitation device is generated by the algorithm based on the classifier, which is capable of detecting user movement intention in real time. The main aim of this study is to prove that the device control algorithm is adapted to the characteristics and necessities of the user through the proposed classifier with high accuracy in hand gesture recognition.

## 1. Introduction

As a result of complex human evolution, the hand is one of the most versatile parts of our body (Craig and Taylor, 1955). As well as giving us the ability to perform several tasks during our daily life, it is also one of the key factors that differentiates humans from other species. Its 27 degrees of freedom provide a mechanism able to manipulate nearly every kind of object.

According to different studies, a system placed in the brain takes the responsibility of hand control (Hirzinger et al., 1998; Biagiotti et al., 2003; Yue et al., 2017). The control system structure is complex and difficult to understand, which hinders the motor function recovery process after a stroke, disease, or disorder.

With the aim to improve the rehabilitation techniques applied to the human hand, several robotic solutions have been proposed over the past 20 years. The main benefit of robotic rehabilitation is that it allows an active interaction between the patient and the rehabilitation system, which is essential in the recovery process (Londoa et al., 2017).

Taking into consideration the interaction between the user and the rehabilitation system, rehabilitation procedures can be classified into two groups:

- Physical interaction (PI): physical contact between the rehabilitation system and the user is needed in order to apply several forces to the patient during the task performance;
- Emotional interaction (EI): the system encourages the user during the process in the absence of physical contact.

PI rehabilitation using robotic systems requires direct physical contact between the robot and patient during the rehabilitation process. External forces provided by the robot help or hinder patient movement. Usually, the first steps of the rehabilitation process are completed with robotic assistance, while in the last stages, the user receives robotic opposition during the rehabilitation tasks.

For instance, Pyk et al. (2008) proposed PITS, a glove-based robotic system with different position sensing devices. In the same way, the NJIT-RAVR structure is a ring gimbal with six degrees of freedom for hand rehabilitation (Qiu et al., 2009). Both systems perform similarly. A glove covers the hand of the patient which, depending on the system, gives more or less freedom to the user. For example, more freedom is given by PITS as its glove is not linked to any structure, whereas the NJIT-RVAR glove is placed in a ring gimbal with a limited number of degrees of freedom. Also, virtual reality is used for displaying several interactive tasks in the systems. Patients must perform the task in contact with the robotic system which has a haptic master able to capture hand movements and translate them into the computer space. The complexity level of the task displayed is adapted to each patient recovery phase.

Several advantages such as creating a safer environment with nearly zero risk of injury, motivating the patient with different interactive exercises, automation of the rehabilitation process, adaptation of the device to different rehabilitation stages, and quantifying the rehabilitation process making it objective are characteristic of PI rehabilitation robotic devices.

EI rehabilitation focuses on different tasks completed in collaboration with a robotic system using one of these three methods: (1) imitation: robot movements are followed by the user, (2) motivation: robot-user interaction using sounds or visual effects encourages the user during the process, and (3) imitation and motivation: combination of (1) and (2) (Maciejasz et al., 2014). In this context, systems such as CosmoBot (Wood et al., 2009) or Ursus (Calderita et al., 2014) are prominent. The process is simple: the robot performs an action that must be followed by the user. At the same time, the system monitors the progress and status of the patient during the recovery process.

Recently, several studies have proposed the use of electroencephalogram (Zhang et al., 2021, 2022) or electromyography (Ahsan et al., 2011; Asif et al., 2020; Pamungkas and Simatupang, 2020) sensors in order to control robotic rehabilitation procedures, because they are able to detect the patient's intention of motion. Electromyography sensors measure the electrical signals originated in the muscles for quantifying its activity. Information such as the activity performed by the muscle or the effort needed to perform the activity could be obtained by surface electromyography (sEMG) analysis.

Specifically, the type of hand gesture performed by a person could be identified using electromyography sensors (Binh et al., 2005; Alsheakhali et al., 2011; Khan and Ibraheem, 2012). sEMG data captured by electromyography sensors contain features that could be extracted in order to train different neural network architectures. Neural networks are used to predict the type of hand gesture executed by the user. For example, Ahsan et al. (2011) used a back-propagation algorithm to train a network architecture for hand gesture classification using sEMG reaching an 88.4% average success rate of identification. Asif et al. (2020) achieved a 92% average classification accuracy for the same issue using a convolutional neural network architecture. Another possibility presented by Pamungkas and Simatupang (2020) is the use of Bayesian neural network architecture for sEMG classification, which was able to reach a 90.61% average accuracy. All of these architectures could be combined in order to obtain higher accuracy rates (He et al., 2018; Asif et al., 2020).

Hand gesture recognition using sEMG is useful not only for rehabilitation issues (knowing the type of gesture is helpful for the patient during the rehabilitation tasks and allows them to correct the application of movement opposition), but also for building artificial hands or robotic structures with the ability to imitate human motions.

Another rehabilitation system which combines physical contact with robotic devices and sEMG detection is AMADEO (Londoa et al., 2017), a platform with five end-effectors where the five fingers can be placed. Several exercises are displayed in a screen while, at the same time, sEMG signals are recorded. sEMG features are used to help the patient during the activity and to evaluate the rehabilitation process.

Although several works address the topic of sEMG signals for hand gesture identification, few works use these identified gestures for hand rehabilitation devices and they usually focus on the mixed gesture identification between the wrist movements and the hand gesture. In this paper, we propose to identify six hand gestures using only finger movements, which will be implemented in a high-level control algorithm for hand rehabilitation with an exo-glove.

Specifically, this study is focused on the implementation of a PI rehabilitation system combined with an sEMG recognition architecture for hand gesture identification. The goal is to evaluate new classifier structures for sEMG recognition. A novel hand gesture classifier for sEMG based on a neural network architecture (a combination between the Bayesian neural network, pattern recognition networks, and layer recurrent network) is developed, which enables the generation of the position of reference for PI rehabilitation devices according to the specific patient movement intention. Results from this study will ultimately provide insights on the feasibility of the neural networks' structures proposed for hand gesture recognition.

Compared to other state-of-the-art solutions, our approach's contribution is:

- The development of a novel neural network architecture based on the Bayesian neural network, pattern recognition networks, and layer recurrent network with 98.7% accuracy for hand gesture recognition.
- The generation of a new algorithm to calculate the position reference for the rehabilitation device according to the user intention of movement.
- Previous contributions could be used in real time. They were tested in a hand rehabilitation device actuated by shape memory alloy (SMA) and developed by our research group.
- A new user-friendly interface was developed for personalized sEMG acquisition, neural network training and verification, and control of the rehabilitation device.

This paper contains five sections. Section 2 presents the methodology with a description of the sEMG data acquisition and processing method, neural network architectures used for gesture classification, and the rehabilitation system: actuator characteristics and design, rehabilitation device, and the high-level control algorithm. Preliminary experimental results are covered in Section 3. Section 4 presents the discussion and Section 5 proposes the conclusions and future works.

## 2. Materials and Methods

### 2.1. Experimental Protocol

A gesture recognition algorithm is proposed with an individual calibration of the neural networks carried out simultaneously, with the objective of using this information in rehabilitation glove control. For this reason, the experimental protocol consists of:

- The sEMG signal acquisition for a new database consisted of 250 samples per each proposed gesture. One sample represents a 300 ms sEMG signal. In total, 1,500 samples were stored.
- The features were extracted from the sEMG windows (this was done during the acquisition process);
- The features for the 1,500 samples were used in the neural network architecture training and offline evaluation process.
- A total of 100 new samples per gesture (in total 600 samples) were acquired and in this case, storing of the output of the proposed classifier architecture and the user gesture occurred at the same time. With this information the confusion matrix was built to evaluate the proposed architecture in the online evaluation process.
- On the last step, the gesture recognition algorithm was connected with the glove rehabilitation device, and the data from sEMG, gesture, tendon positions, and reference were stored.

### 2.2. Proposed Hand Gesture Identification

**Figure 1:**
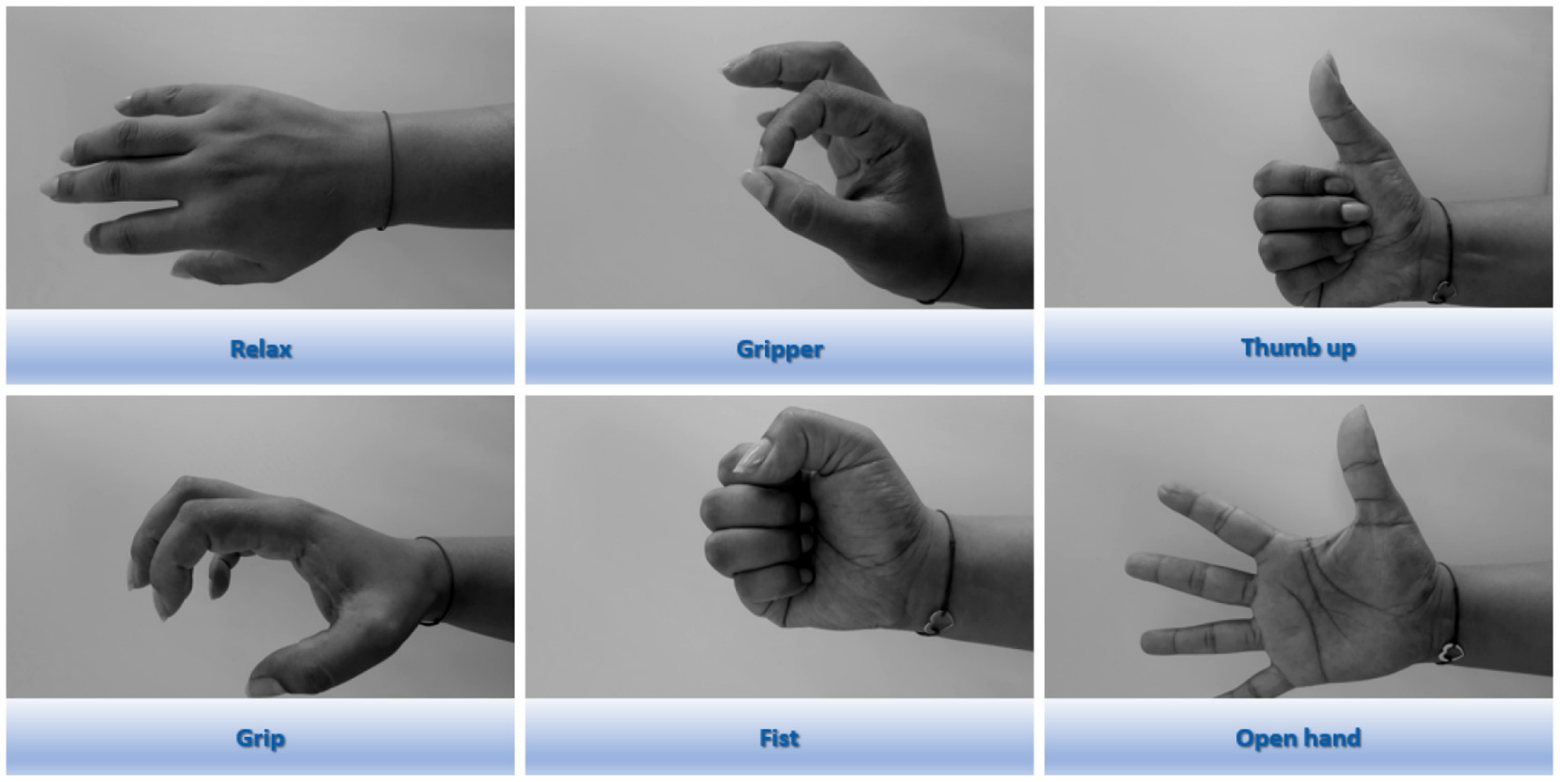
Hand gestures performed during sEMG signal recording.

sEMG data are used for hand gesture identification. Considering the hand movement on daily activities and the consecrated hand rehabilitation movements, six hand gestures are proposed for the identification:

- Relax, lack of user movement;
- Gripper (pinch), tap the thumb with the index finger;
- Thumb up, thumb extension;
- Grip, replication of object holding;
- Fist, close the hand;
- Open hand, finger extension;

The proposed classifier must be able to identify these gestures regardless of the user's hand: left or right, and based on this, must generate the reference for the rehabilitation glove. Right hand gestures are shown in Figure 1.

sEMG data are collected using the Thalmic Labs Myo Gesture Control Armband (Huitzil-Velasco et al., 2017). The armband features eight sEMG sensors with a stream rate of 200 Hz. According to Merletti and Parker (2004); Konrad (2005) almost all of the EMG signal power is located between 10 and 250 Hz, and considering the Nyquist-Shannon sampling theorem, the amplifier device band will need to be set to 500 Hz or higher. In this case, a part of the signal will be lost, the armband frequency being limited to 200 Hz. sEMG sensors are placed over the forearm giving information of the activity of the arm muscle groups responsible for hand and wrist movements. As a non-invasive method, the accuracy of the sEMG acquisition process depends on well-known factors such as electrode position, skin factors, ambient noises, and movement noises.

Also, it is usually good practice to rectify sEMG a priori only considering its absolute value. Note that this change can affect other characteristics, like the frequency which will be doubled.

Another interesting process is sEMG filtering. It is a good technique for noise reduction as unwanted harmonics are removed. Several studies use upper cutoff frequency filters (around 500 Hz). In general, notch filters with lower cutoff frequencies between 10 and 500 Hz are common, although it depends on the study and the limb analyzed. Butterworth architecture filters are the most common. Balbinot and Favieiro (2013) used a 60 Hz notch filter to remove the noise characteristic of the power line (in Europe it would have been 50 Hz).

Another possibility is to normalize the sEMG amplitude as it prevents noise variations with no influence on the classification (Konrad, 2005).

After these previous steps: rectification, filtering, and normalization, sEMG can be more easily interpreted and classified. In this work only the rectification process was implemented because the signals were filtered and normalized by the Myo SDK (Tomaszewski, 2016).

**Figure 2:**
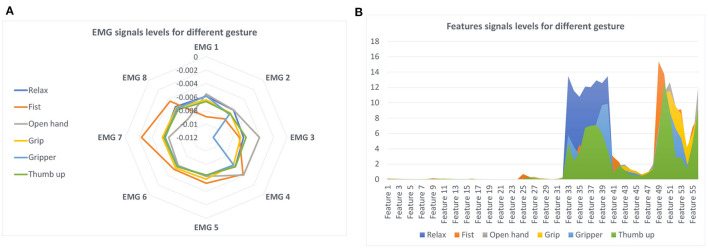
Gesture according with the sEMG signal and features: **(A)** sEMG signals; **(B)** signals features.

The sEMG level for each gesture is presented in Figure 2A. After the feature extraction process (Figure 2B), an average of 1,000 samples from one of each electrode is acquired for each proposed gesture. The fist gesture can be easily distinguished from the open hand gesture, however, other gestures like the gripper and thumb up gestures are more similar. Moreover, when the average of several signals is plotted on the graph (Figure 2A), a certain signal segment for a short time can negatively influence the classification.

Results demonstrate that sEMG signal segmentation and feature extraction are needed. There are two main techniques for signal segmentation: adjacent segmentation and overlapping segmentation. The adjacent segmentation technique was selected. In this approach, sEMG data are split into adjacent windows. According to Oskoei and Hu (2007), a real-time classification is considered when the length of the segment lasts less than 300 ms, but the longer the segment, the more accurate the classification of the gesture. For this reason, segments were fragmented into windows with a fixed length of 300 ms. In each window, seven time-domain features were calculated: (1) mean average value (MAV), (2) root mean square (RMS), (3) variance (VAR), (4) signal strength indicator (SSI), (5) zero-crossing (ZC), (6) wavelet transform (WL), and (7) side scatter (SSC). These represent 56 values extracted from each window. Values 1–8 represent the first feature MAV for each electrode (8 electrodes), from 9 to 16 represent the second feature RMS for each electrode, and so on. Previous studies used these features for classification (Phinyomark et al., 2012; Phinyomark and Scheme, 2018; Barioul et al., 2019; Wu et al., 2020; Khushaba et al., 2022).

Features from 100 segments for each gesture were extracted where the mean value is represented in Figure 2B. Although the first features do not seem to be relevant, the last features showed notable differences in the gesture recognition. Similarly to sEMG, noticeable differences could be observed when observing the fist and hand open gestures; but the gripper and thumb up gestures could be easily confused.

**Figure 3:**
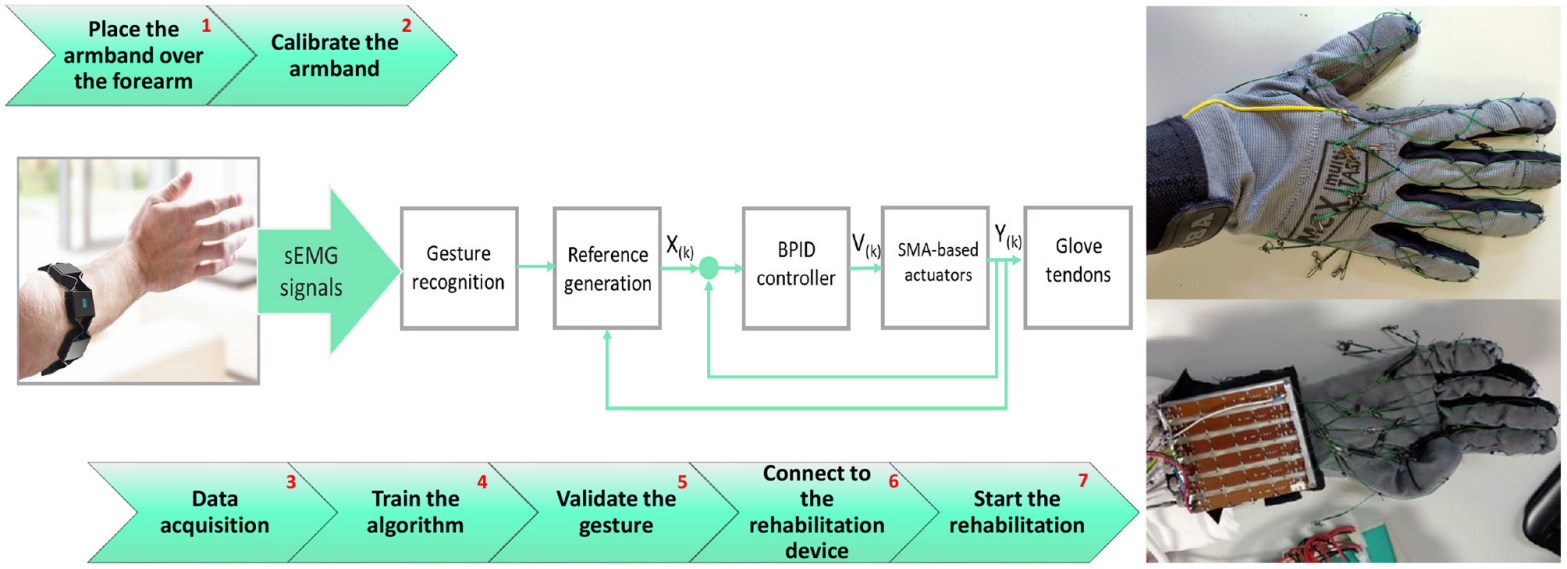
Overview of the proposed rehabilitation system.

These characteristics will be used as an input for the proposed classifier for gesture recognition. An overview of the proposed rehabilitation system can be seen in Figure 3. After a minimal set-up consisting in placing the armband over a forearm and its calibration (steps 1 and 2 from Figure 3), the data acquisition process, training the network architecture, and the validation of gesture recognition are necessary (steps 3, 4, and 5). The process continues with the control algorithm which generates the tendon references according to the gesture recognition and ends with glove rehabilitation device connection, and then the rehabilitation therapy can begin (steps 6 and 7).

### 2.3. Classifier Architecture

**Figure 4:**
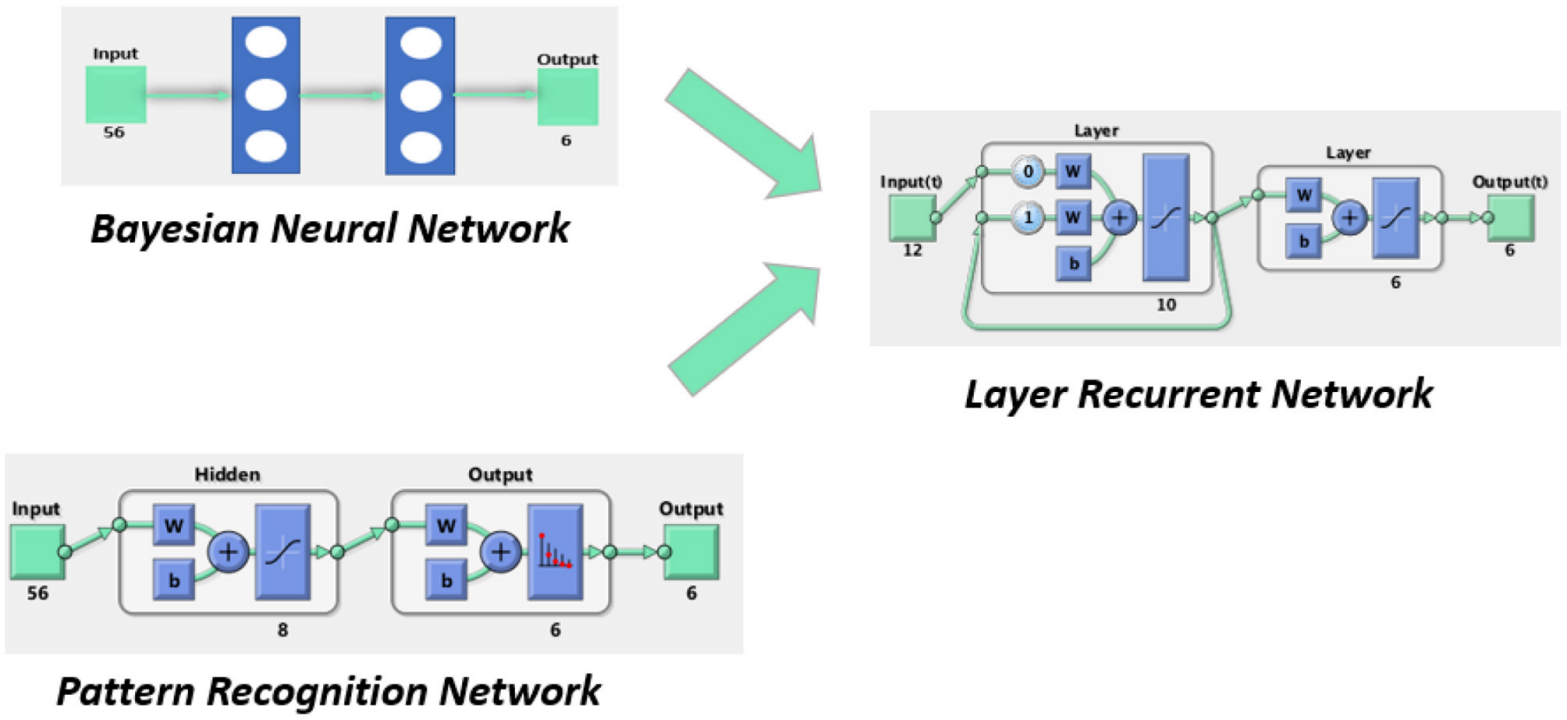
Proposed neural network architecture.

During the classification process, each hand gesture presented in Section 2.2 was related with static position information given by the sEMG features. The proposed classifier in this work is based on three sub-architectures: a Bayesian neural network (BNN) in parallel with an artificial neural network (ANN) in which the results are connected in series with a layer recurrent network (LRN). The final gesture classification is the result of the LRN. The proposed architecture can be seen in Figure 4. Each supervised architecture was configured and trained as follow:

- ANN: A feedforward network with the ability to classify different inputs according to target classes. The target data for pattern recognition networks consist of arrays of all zero values except for a 1 in element *i*, where *i* is the class it represents. In this case, there is a 56-feature input array whose target is represented by a 6-element vector, each one for a specific gesture recognition. The proposed architecture contains two layers; the first hidden layer of eight neurons and the output layer with six neurons corresponding to the six gestures. Weights and bias parameters of the neurons were adjusted using the scaled conjugate gradient (Hestenes and Stiefel, 1952) and its performance was evaluated with cross-entropy. Like BNN, ANN was trained with 250 samples but in this case the data were divided into 70% for training, 15% for testing, and another 15% for validation. The data for validation and testing were not used during the training process.
- BNN: A probabilistic classifier based on the naive Bayes assumption (predictors are independent of one another within each class) (Martinez-Arroyo and Sucar, 2006). The network, built with the classification learner app in Matlab 2020b (The MatWorks, Inc., 2021), uses a kernel distribution because the Gaussian distribution often results in error due to the non-Gaussian distribution of the sEMG features. For offline training, 250 samples were used from each hand motion and a five-fold cross validation was employed during the training process: each set of five samples was divided into four samples for training and one sample for validation.
- LRN: This architecture presents two layers: a hidden layer with 10 neurons that receive information from the past by taking into account the previous results for future predictions and a output layer with 6 neurons; a vector of 6 positions, one for each gesture. LRN input results from the output of the first two networks combined in a 12-element array. LRN remembers a past sample structure by using a feedforward network, being capable of analyzing sequential data structures, such as consecutive hand gestures. LRN is trained using 250 samples using the Levenberg-Marquardt algorithm (Levenberg, 1944) while its performance is tested with mean squared error. LRN receives 12 inputs obtained from 6 inputs of the BNN prediction over these 250 samples and another 6 inputs produced by ANN over the same 250 samples. Similarly with the ANN training process, the data were divided into 70% for training, 15% for testing, and another 15% for validation.

### 2.4. User Interface

Though the armband device must be placed in a certain position on the forearm, the possibility that the electrodes are placed over the same muscle fiber in two different rehabilitation sessions is very low. For this reason, a new personalized dataset was created before each rehabilitation session from the user sEMG (forearm muscles sEMG) data. During this process, features are extracted from each sEMG segment and a file with the name of the motion and the number of the sample is stored in a folder called Dataset. Each sample contains a 56 × 1 double array, from the seven features for each sEMG segment. For building the neural network architecture, 56 × 600 feature samples were collected for each motion, resulting in a database with 56 × 3600 samples. Depending on each supervised architecture, the whole dataset is split randomly into 70% for neural network training, 15% for validation, and another 15% for tests for the ANN and LRN, and using the five-fold cross validation for BNN.

**Figure 5:**
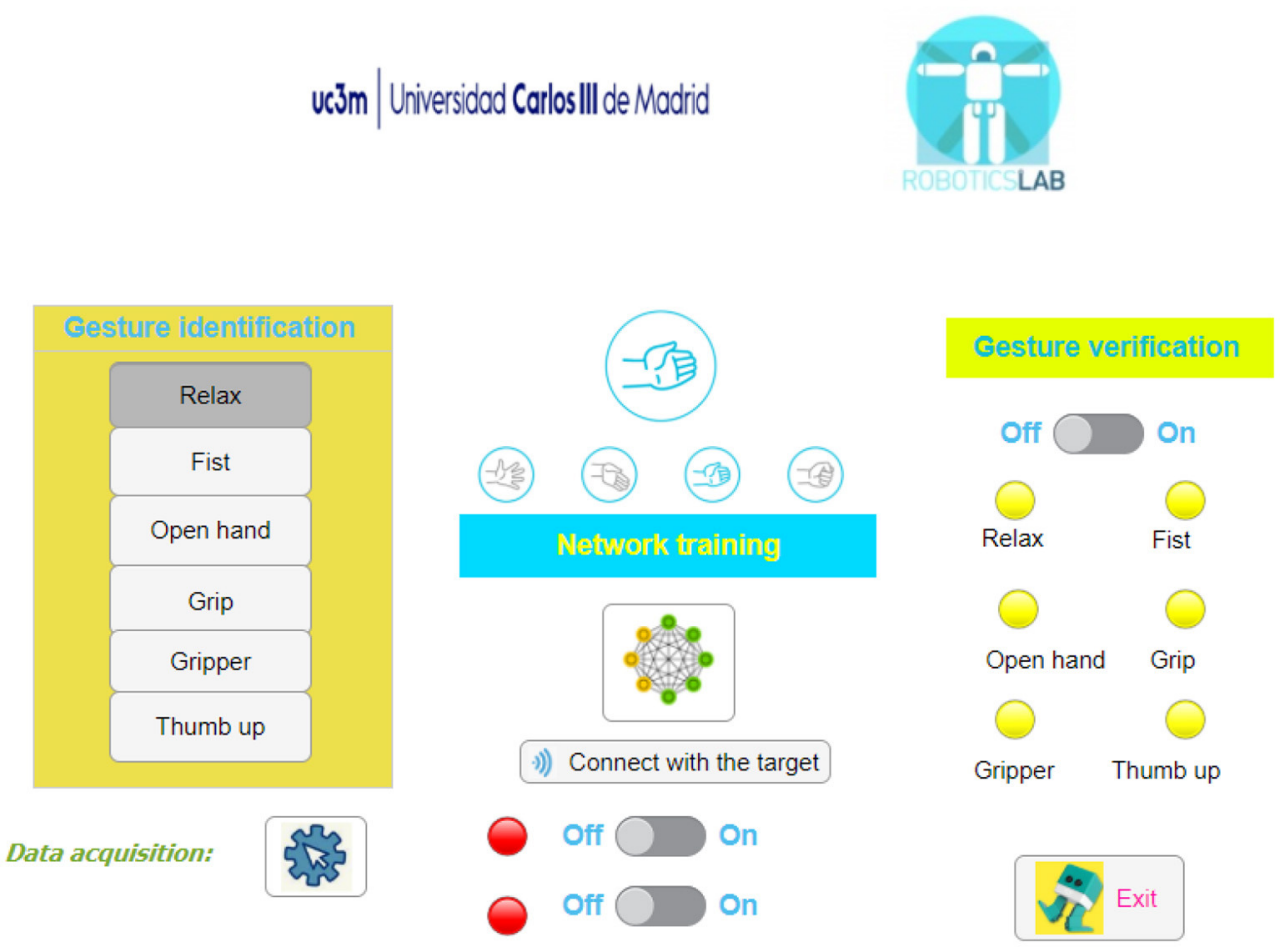
User interface for the developed application.

To automate and speed up this process, a user interface is developed in Matlab 2020b (The MatWorks, Inc., 2021) as an intuitive way of following the seven steps detailed in the pipeline in Figure 5. The interface is shown in Figure 5.

After the armband is placed, the user is asked to perform each gesture in order to create the personalized database. After that, the button—network training—automatically trains the Bayesian classifier. For gesture validation, the switch button is turned on and the lamp of the recognized gesture is colored green while the other lamps are red. The bottom switch buttons are used to connect the rehabilitation device and start the therapy.

### 2.5. Rehabilitation System

Restoring the hand function after spinal cord injury (SCI), cerebral vascular accident (CVA), or different musculoskeletal disorders represents a challenging issue. Rehabilitation gives the user the possibility to recover the ability to perform daily life tasks. Recently, several exoskeleton devices have been proposed for hand rehabilitation over the last years, but most of them focused on soft and low-cost designs, offering a passive rehabilitation without taking into account user movement intention. The rehabilitation device used in this paper is an SMA-actuated glove. It is a wearable device, with low weight and noiseless performance due to the actuators' characteristics.

#### 2.5.1. SMA-Based Actuator

SMA is an alloy, commonly Ni-Ti, which has the property to deform when it is cold and recover its pre-deformed shape (“memory”) when heated. This process takes place between the two transformation phases: martensite at low temperature and austenite at high temperature. To achieve the necessary transformation temperature, electrical energy is transformed into thermal energy thanks to the Joule effect. Due to the shape memory effect (SME), thermal energy is transformed in mechanical work. The SMA-based actuator used in the proposed rehabilitation device is based on Copaci et al. (2019a), and it is composed by:

- Bowden cable: a metallic spiral covered with a nylon sheath. It has the property of SMA wire force transmission, achieving the flexibility property. Also, it helps in wire heat dissipation in the cooling stage.
- Polytetrafluoroethylene (PTFE) tube. The isolator is placed between the SMA wire and Bowden cable and is able to resist more than 250°C. It is considered as a solid lubricant because it decreases the friction in the SMA wire.
- SMA wire. Flexinol wire from the Dynalloy company was used (DYNALLOY, Inc., 2020). With a diameter of 0.51 mm, it applies a force of approximately 34.91 N. The wire activation temperature is 90°C where an ~4% displacement of the wire length is reached.

According to the necessary finger tendon displacement (~8 cm), 2 m of SMA wire is needed. Due to the actuator flexibility, it can take on the human body shape and can possibly be used to guide the user.

#### 2.5.2. Glove Rehabilitation Device

The rehabilitation glove is presented in Figure 3. A strong but comfortable mobility glove is used to withstand the strength of the cables (tendons) without tearing and, at the same time, allowing natural movements to the fingers. Twelve tendons were guided/routed over the glove to generate the flexion/extension movement in each finger contraction (five tendons for extension and another five for flexion) and another two to help in the thumb opposition movement.

Glove tendons are connected with SMA-based actuators through a sensor box composed of six rails where a small cylindrical piece, which connects the SMA wire with the tendons, could be moved when the actuator is enabled. The same piece is connected to a Bourns PTA Potentiometer, PTA6043-2015DPB103 which is able to measure each tendon displacement.

In addition to the six position sensors, we used electronic hardware of the glove rehabilitation device including a microcontroller and a power circuit essential in controlling the SMA-based actuators as feedback for the controller loop.

The electronic power circuit for SMA wires is based on MOSFET transistors which can be activated by pulse width modulation (PWM) generated by the controller. MOSFET transistors open and close the circuit with a power supply for actuators. The control hardware architecture can manage six different actuators; in this case each actuator was an SMA wire. The whole architecture has been developed by our research group.

The controller board is based on the STM32F407 Discovery kit (STM, 2021), from STMicroelectronics, which is programmed using Matlab/Simulink^®^ (Caballero et al., 2016). The board manages signals from the sensors, executes the control actuator algorithm, and generates the required PWM signals. The low-level control used for this device is based on a bilinear proportional integral derivative (BPID) controller, developed by the research group presented in Villoslada et al. (2015); Copaci et al. (2019b).

The identified hand gesture together with the sensors positions signals from the rehabilitation device and generates six reference signals building a representation of the user's movement intention for each finger (two references for the thumb). In this way, an active reference is generated involving the patient undergoing rehabilitation therapy, leading to a faster recovery.

### 2.6. Reference Generation

According to the rehabilitation device structure, these six references are duplicated with the opposite ones to provide the inputs for the low-level control algorithm (six references for the flexion actuators and six references for the extension actuators) for the antagonistic movement. However, the current device only presents a boxed sensor and it can only be tested in one of the movements, for example the flexion movement.

The entry algorithm from the sEMG data is captured by the glove tendon movements as can be seen schematically in Figure 3. *X*_(*k*)_ represents the reference generated by the high-level algorithm, *V*_(*k*)_ represents the control signal (PWM generated by the microcontroller for the electronic power circuit), and *Y*_(*k*)_ represents the position of the actuator signals from the position sensors.

The reference generation block from Figure 3 receives the recognized gesture as input, a six-number array from 0 to 1, where each number represents the probability of being the gesture performed. The maximum value of the array represents the gesture predicted. Apart from the gesture array, a six-position array is generated. In this case, the position represents the maximum actuator reference reached according to the gesture. Using this six-position array, the actual actuator positions, the actual reference positions, and two adjustable increments (six references in real time for the actuators) are generated. Increments are used to change the reference speed and the finger movement speed. A higher increment is required to move from the current reference to the current position of the actuator, and a slower one is required to generate a smoother reference when we are close to the current position (used when the reference wants to be followed, during the gesture performance).

## 3. Results

### 3.1. Offline Classifier Results

The three architectures presented in Section 2.3 were offline-evaluated with a database with 1,500 samples (250 features for each gesture). The results obtained with each architecture are detailed here.

**Figure 6:**
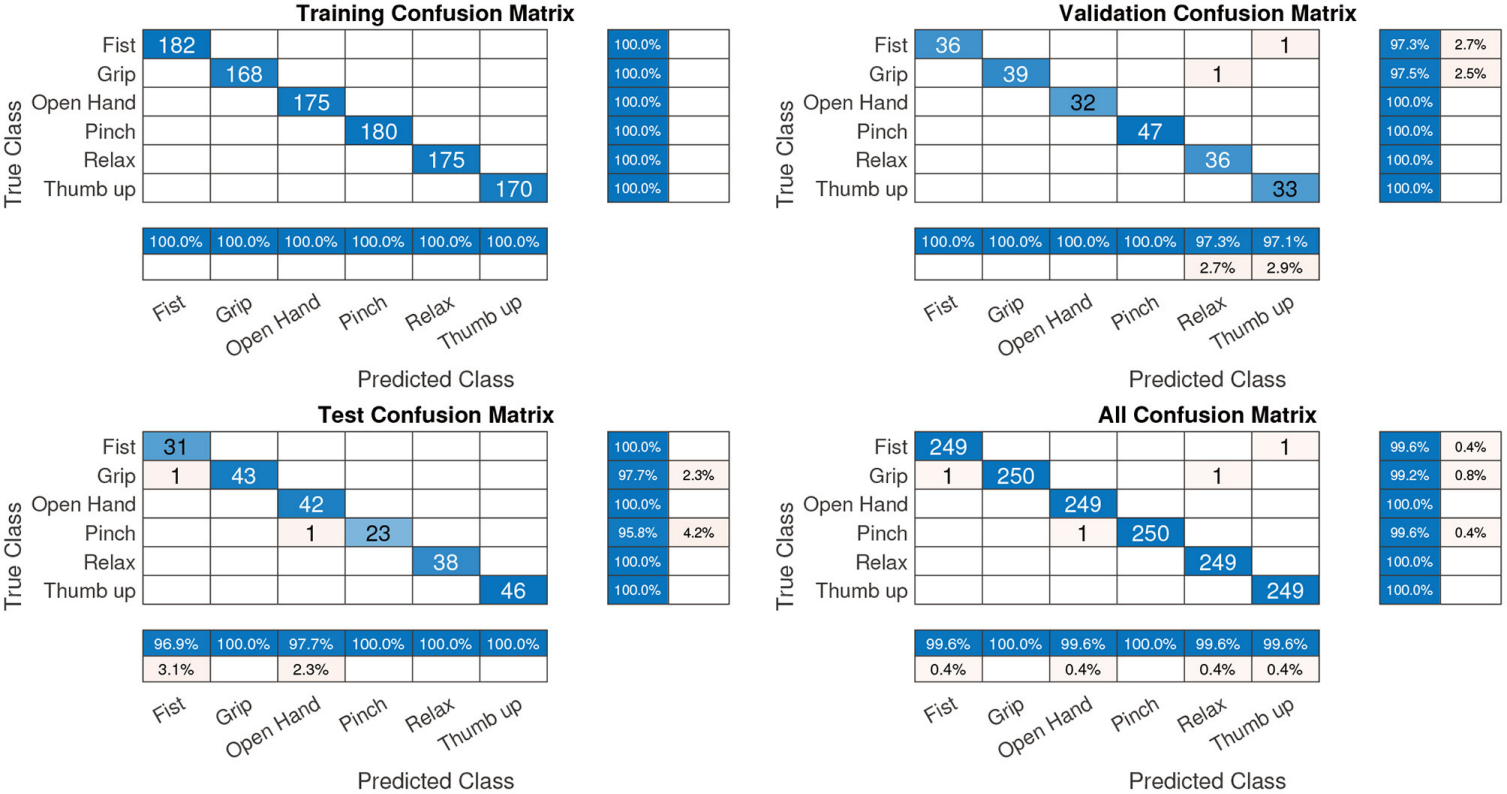
Confusions matrix of the pattern recognition network—offline. All confusion matrixes had an accuracy of 99.7%.

For the ANN architecture, the samples were split into three groups: 70, 15, and 15% for training, validation, and testing, where the validation and test data were not used in network training. After the network training, the confusion matrix of each group was determined as shown in Figure 6.

In Figure 6, the diagonal cells (in blue) correspond to observations/gestures that are correctly classified. The off-diagonal cells correspond to incorrectly classified observations. In the validation and test confusion matrix, as can be observed, the gesture recognition is 99.1%, where the predicted percentage or output corresponds to the rows and the target class corresponds to the columns. The last row represents the normalized row which summarizes the percentages of correctly and incorrectly classified observations for each true class. The last column, a normalized-column summary, displays the percentages of correctly and incorrectly classified observations for each predicted class. For example, in the confusion matrix test (Figure 6), one fist gesture was classified as a grip gesture. In this case, the predicted fist gestures were classified correctly with 100% accuracy (last column, first row) but the predicted grip gestures were classified correctly with only 97.7% accuracy (last column, second row). In the last row, first column, 96.9%, of the fist gestures were correctly classified for the true fist class.

**Figure 7:**
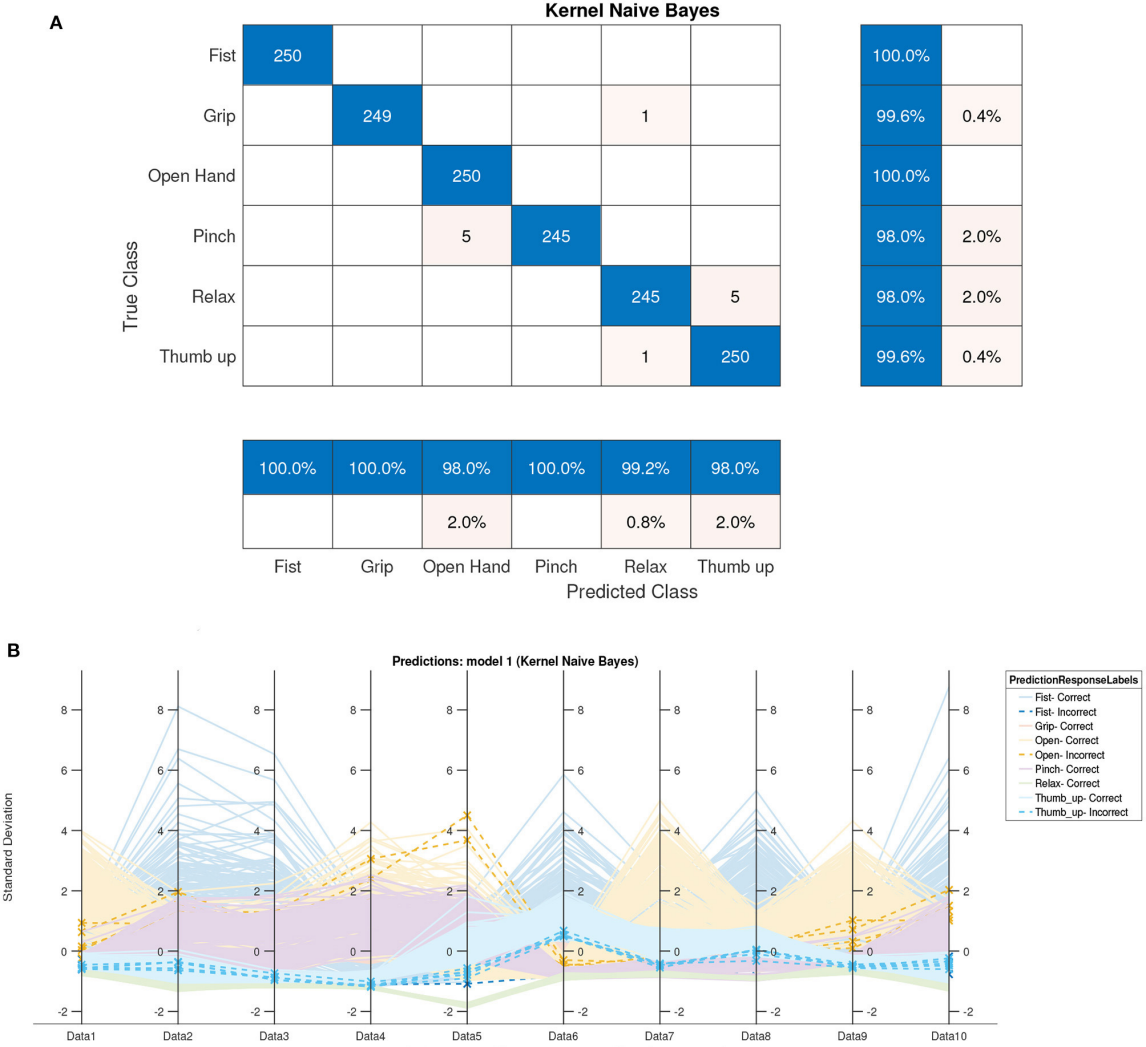
Naive Bayes offline classification results. **(A)** Confusion matrix of naive Bayes (accuracy 99.2%). **(B)** Parallel coordinate prediction of naive Bayes.

The kernel naive Bayes classifier achieved 99.2% accuracy. The confusion matrix is presented in Figure 7A where five samples from the pinch gesture were classified as an open hand gesture, five samples for the relax gesture were confused with the thumb up gesture, and one sample of the grip and thumb up gestures were classified as the relax gesture. Figure 7B presents the parallel coordinate prediction where each sample is represented (correct classified—continuous line and incorrect classified—dotted line).

With the results of the two architectures and the output target, the LRN architecture was trained. Similar with the ANN case, the data were split into three groups before the network training. The best validation performance was obtained after 57 epochs of training, with a value of 0.00144.

### 3.2. Online Classifier Results

**Figure 8:**
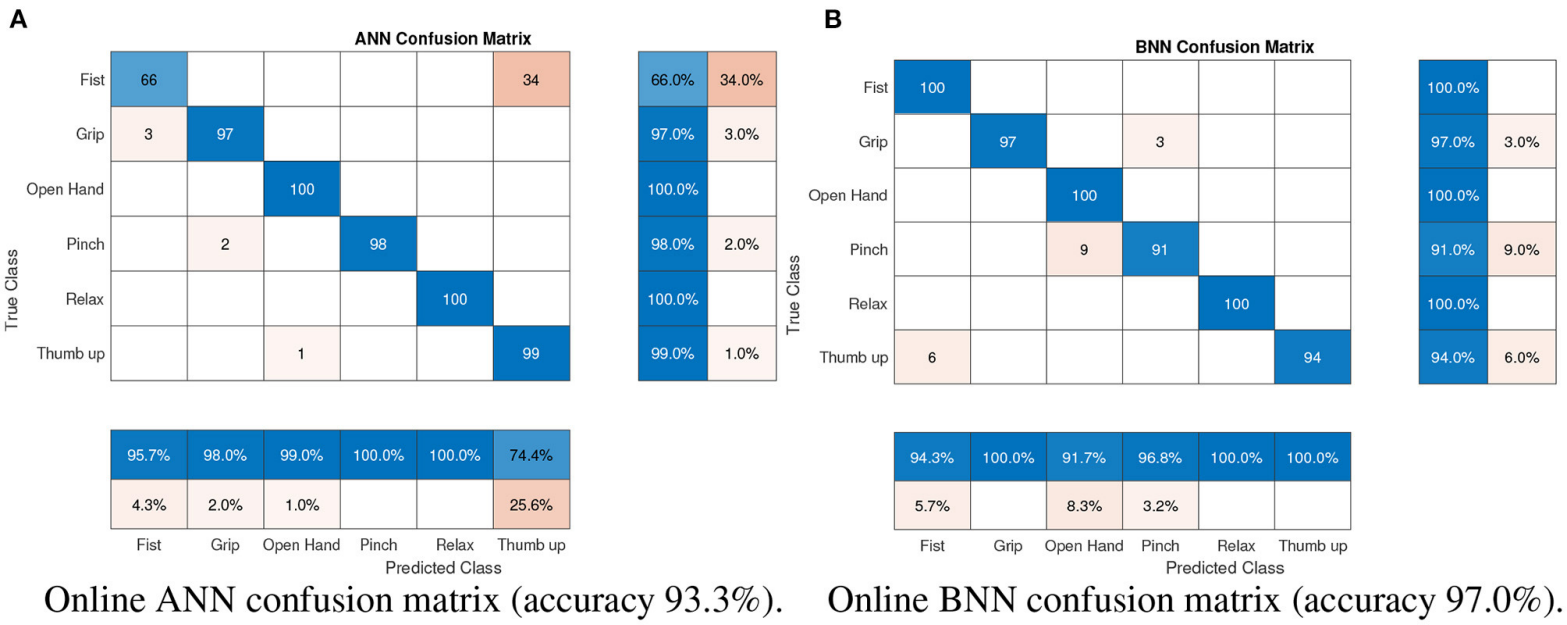
Confusion arrays of the two classifiers: **(A)** pattern recognition network; **(B)** Bayesian neural network.

For online validation, with the trained architectures, a new dataset was stored containing the outputs of each architecture and target gesture. According to these data, the confusion matrix of each architecture was built. Only 100 samples of the training database were used for the final three-network architecture testing. The ANN and BNN classifier responses are represented in the confusion arrays in Figure 8.

**Figure 9:**
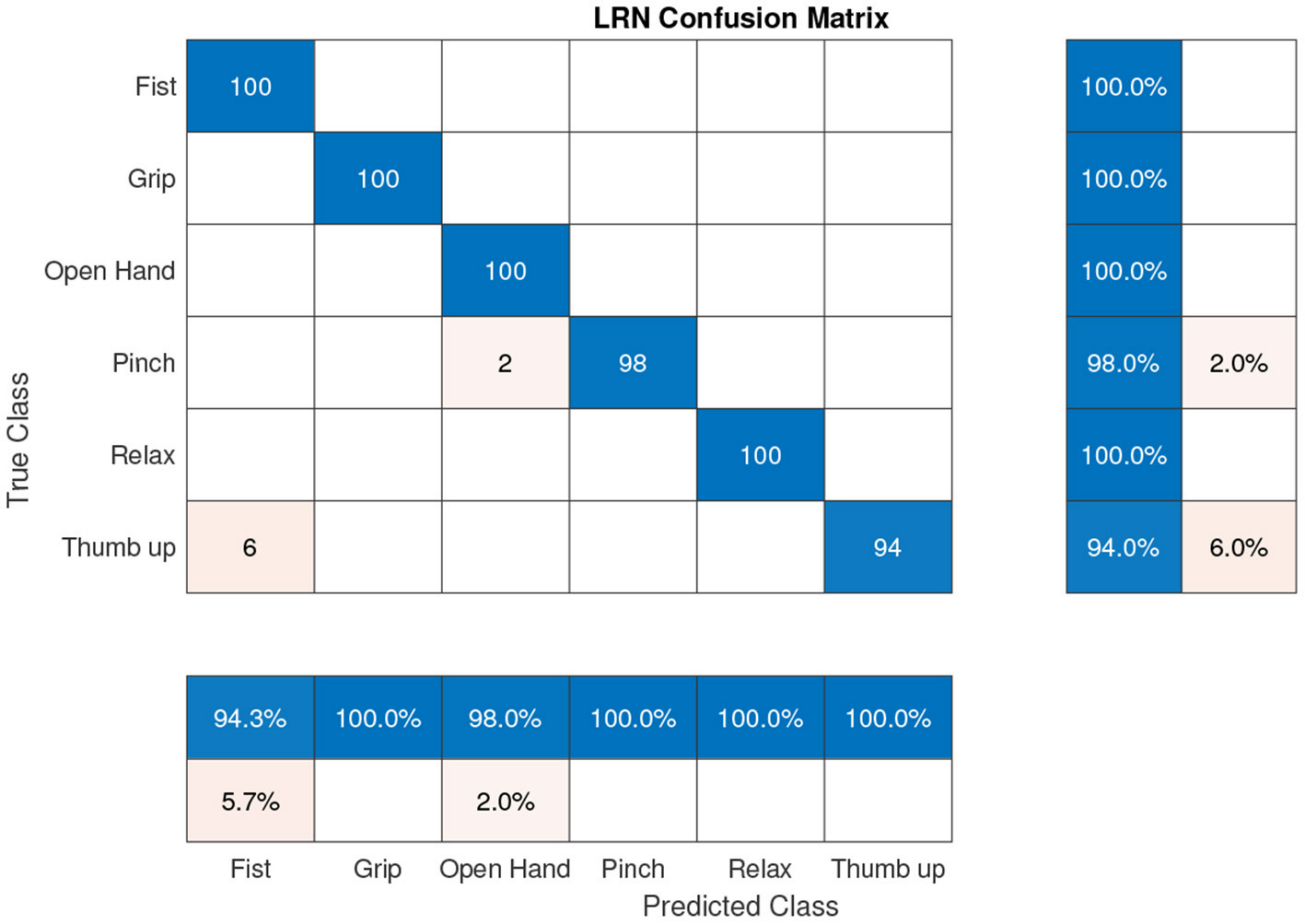
Online LRN confusion matrix (accuracy 98.7%).

According to the results presented in Figure 8B, gesture classification with BNN is more accurate with a 97.0% hit rate compared with the ANN architecture which had a 93.3 % hit rate (Figure 8A). Although the BNN architecture presents better results in general, the ANN architecture for a specific gesture presents a good classification, for example the classification results of the pinch gesture. To reduce gesture confusion, a combination of both architectures was proposed using another neural network, LRN, which also takes into account the past classification gesture. The online results of this LRN architecture can be seen in Figure 9.

Results presented in Figure 9 show that the proposed hand gestures can be classified with a precision of 98.7%. The three-neural network architecture increases the final hit rate in comparison with previous BNN or ANN approaches. The neural network was tested with different users obtaining a percentage between 92 and 99%.

The whole hand gesture classification process takes approximately 0.12 s while sEMG window segmentation lasts 0.3 s meaning the whole classification process takes less than 0.5 s. Time consumption depends on the computer components measuring the times. We utilized an Intel (R) CORE (TM) i7-5500U CPU @ 2.40 GHz and 16 GB RAM in Windows 10 64bits. A specific database for a new user and all neural network training with 100 samples for each gesture (in an automated way with the interface) requires 3–5 min, depending on each user experience.

### 3.3. Gesture Recognition and Reference Generation

Gesture recognition and reference generation evaluation according to the user's movement intention are performed using a new healthy subject who has not tested the Myo Armband with this algorithm before. The rehabilitation device is placed in a test bench over the forearm, below the elbow, and the process starts with data acquisition. Overall, 100 samples were recorded in the database for each gesture. Neural networks were trained with these data. Finally, the user must perform some gestures to test the gesture recognition system. The system is connected to the rehabilitation device.

**Figure 10:**
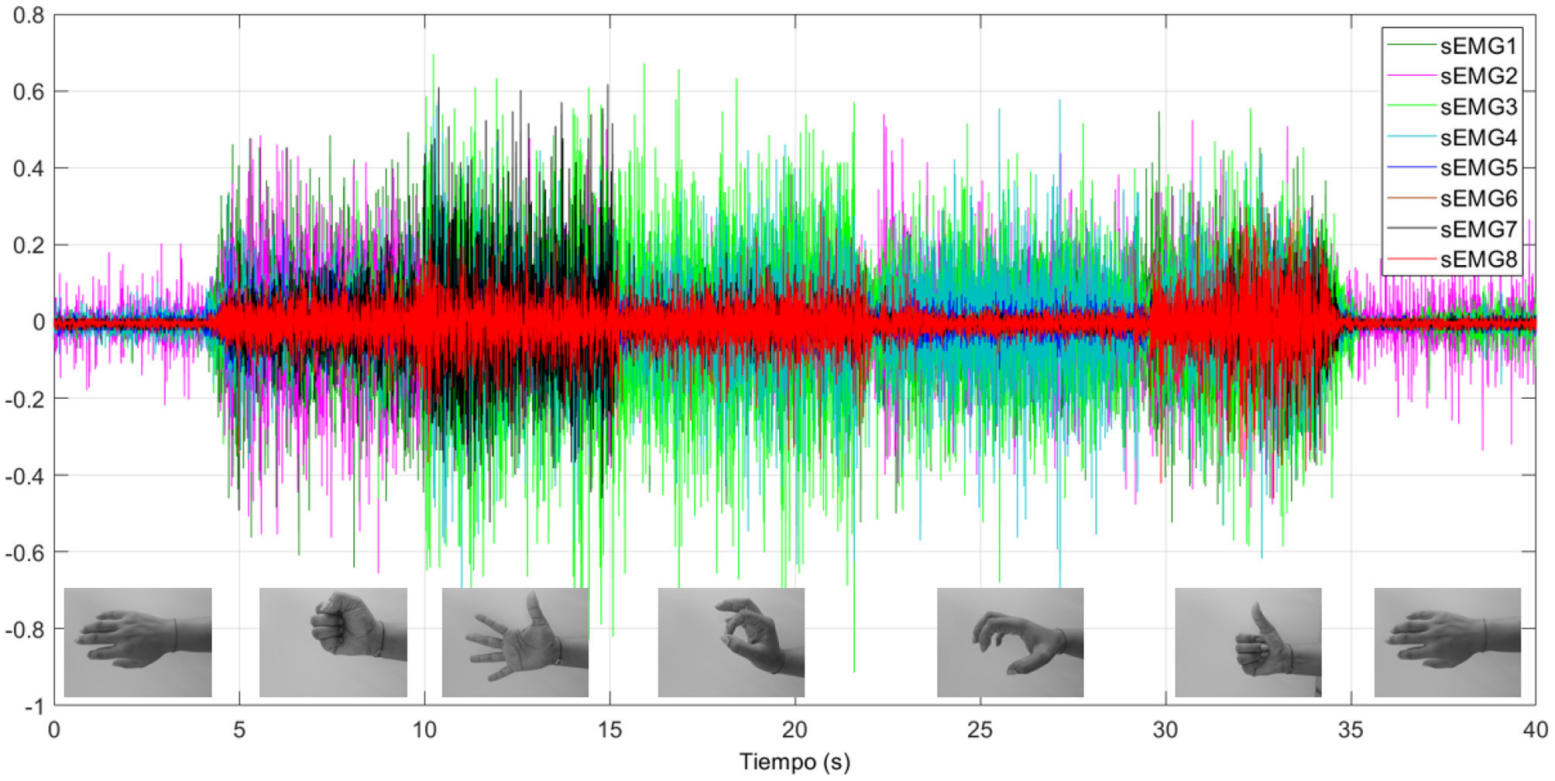
sEMG signals for the eight sensors for the gestures tested.

**Figure 11:**
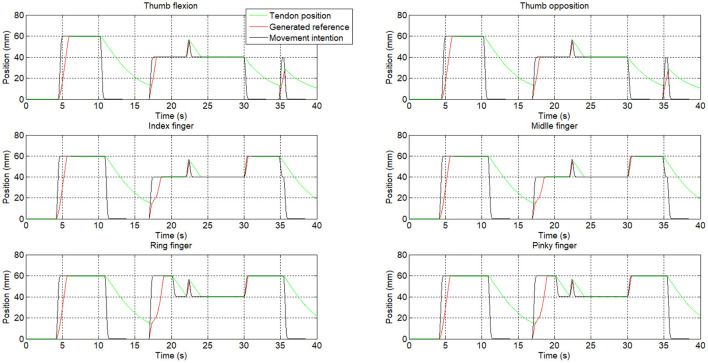
Position response of the rehabilitation device.

Some gestures are recorded for 40 s. sEMG data from eight sensors are presented in Figure 10. Signals are related to:

- from *t* = 0 s to *t* = 4.5 s, the hand is relaxed. Actuators are off and there is free movement of the hand.
- from *t* = 4.5 s to *t* = 10.25 s, the fist gesture is performed. The actuator step reference intention (movement intention represented in Figure 11) for each finger is 60 mm, presenting all the fingers in a flexion movement for closing the hand. The reference intention is not the final reference which is the actuator input. The input reference to the actuators is composed by the intention reference helped by the increments.
- from *t* = 10.25 s to *t* = 16.76 s, the hand open gesture is presented. In this case, the finger intention reference is 0 mm, so the rehabilitation device structure, which only presents actuators for flexion, is left to free the hand and is opened by the user.
- from *t* = 16.76 s to *t* = 20.18 s, the gripper gesture is shown, in which the thumb flexion, thumb opposition, index finger, and middle finger have a step reference intention of 40 mm while the last two fingers (ring and pinky) have a reference intention of 60 mm to simulate the gripper gesture position.
- from *t* = 20.18 s to *t* = 29.97 s, the user performs the grip gesture. All the actuators have a step reference intention of 40 mm.
- from *t* = 29.97 s to *t* = 35.46 s, the thumb up gesture is found. The thumb flexion and the opposition actuators have a step reference intention of 0 mm while the rest of the fingers have a step reference intention of 60 mm.
- from *t* = 35.46 s to *t* = 40 s, the hand relaxed gesture finishes the test.

Neural networks can clearly identify each hand motion achieving accuracy in each gesture recognition. According to Figure 11, gestures are clearly identified except for a little instability at *t* = 22 s. Also, the transition between gestures could be identified as a different gesture. For example, at *t* = 35 s, the user switches from the thumb up gesture to the relax gesture, but in this transition, a grip gesture was identified.

According to the movement intention (black signal in Figure 11), the actuator reference (red signal) is generated. The movement velocity (the slope) could be modified thanks to the increments which can be personalized. The green signal represents the actuator position, which is related to the finger position. For example, the error between the reference and the actuator position between *t* = 11 to *t* = 17 s is due to the actuator behavior (the SMA-based actuator needs to be cold to expand).

## 4. Analysis and Discussion

The proposed classifier achieved 98.7% precision with the gesture classification, which is a promising result. In this work, according to the final application (generating the rehabilitation glove reference) only finger movements were considered due to the fact that the rehabilitation device did not permit wrist movement.

It is difficult to compare the state-of-the-art methods with our data acquisition and network training method. In this work the process consists in acquiring data (600 samples), training the proposed architecture, and after that, starting the rehabilitation therapy. This personalizes the classifier for a specific user. This approach has the disadvantage that the user spends 3-5 min acquiring the data and training the classifier but offers good accuracy in gesture classification. If the classifier is not retrained for a new user, the accuracy of the classifier decreases depending on different characteristics: how the armband is collocated, muscles route, and so on. The literature does not personalize this process using a dataset which contains samples from different subjects. With this point of view, they do not spend time in the training process with the user as the neural network is already trained, which is more comfortable for the user. In contrast, the classification results are poorer.

Compared with the literature (although gestures are different), using the same device with feedforward neural networks, 90.1% accuracy is achieved for the fist, wave-in, wave-out, open, and pinch gestures by the system proposed by Benalcazar et al. (2018). In our case, with the six gestures (relax, fist, grip, gripper, open hand, and thumb up), the feedforward (pattern recognition network) method achieved 93.3% accuracy online. In Ahsan et al. (2011), they built a feedforward network with one hidden layer (10 neurons with tangsig activation function) and one output layer (4 neurons with purelin activation function) for left, right, up, and down gestures, presenting 89.2% precision. Likewise, the architecture proposed by Ahsan et al. (2011) was replicated and has been tested with our dataset achieving 93.8% accuracy. Asif et al. (2020) developed a convolutional neural network tested in real time with 18 subjects for close hand, flex hand, extend hand, and fine grip gestures, reaching 83.7 ± 13.5%, 71.2 ± 20.2%, 82.6 ± 13.9%, and 74.6 ± 15% hit rates, respectively. This last case could not be replicated as they used a deep learning architecture.

**Figure 12:**
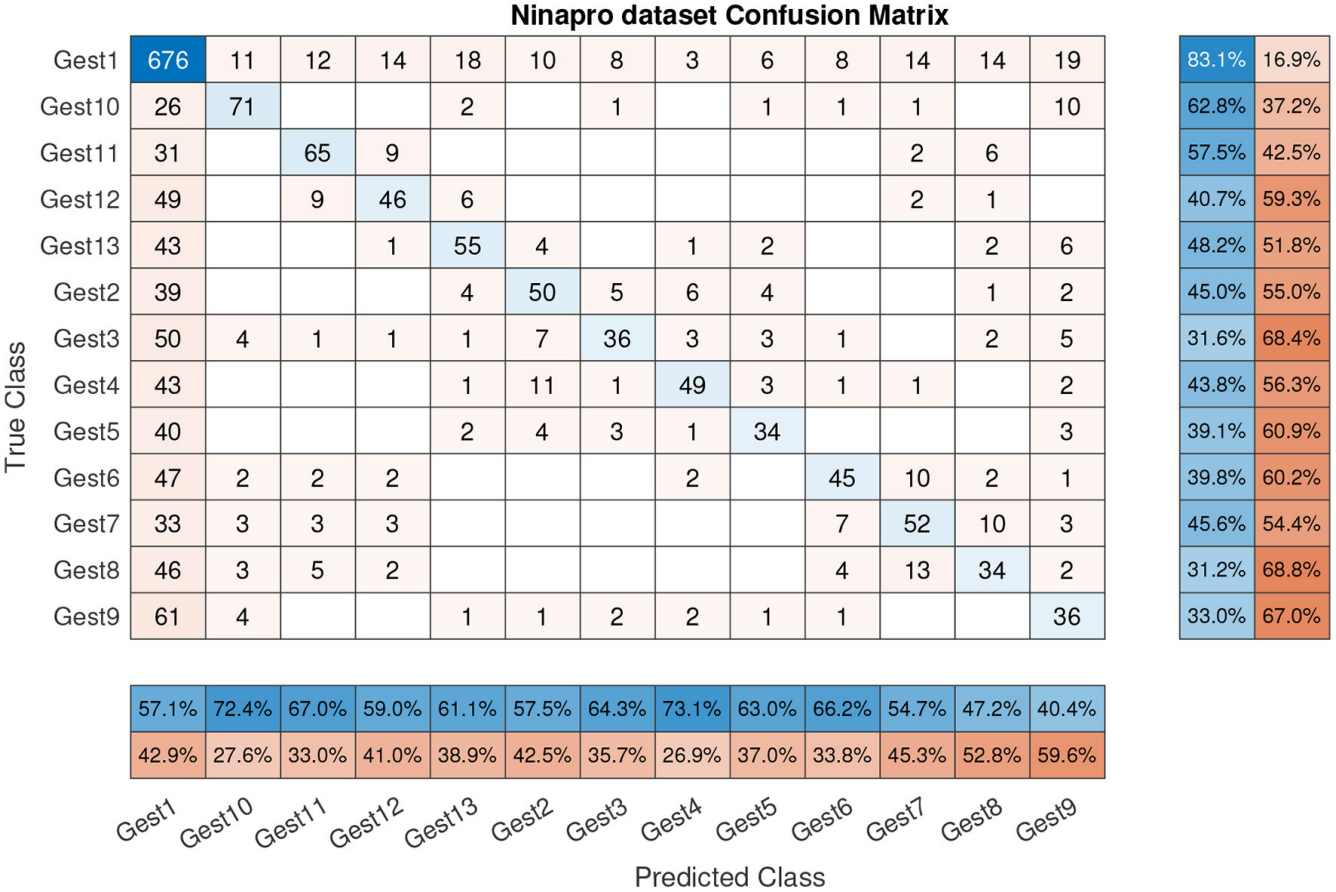
Confusion matrix with NinaPro dataset (58.5% accuracy).

A well-known database, NinaPro Database 5 (Pizzolato et al., 2017), was used to test this architecture. The sEMG features of this dataset were acquired with two Myo Armbands, in total 16 channels. For this test only the first 8 channels corresponding to the first Myo armband were selected for more similarity with this work. The presented results were obtained with only one Myo armband. Before the test, the data were segmented, and from each segment the 56 features were extracted. In parallel, the target gesture was stored to be used for supervised training. This database consists of 13 gestures, for this reason the proposed architecture was modified according to this at 13 outputs. In this case the accuracy result for the 13 gesture classification was 58.5%. The confusion matrix of this dataset can be seen in Figure 12. The biggest confusion was between the gestures (gesture 2 to gesture 13) and the relaxed gesture. This can be influenced by the target gesture data (a scalar value) which were created for each 300 ms segment (60 samples). This target data were set with the stimulus value from the dataset, the value of the sample *n* = 31. In our case, when the database is created for a new user, we store each gesture separately in parallel assigning the target value.

Similar research with the NinaPro database obtained an accuracy between 42.47 and 68.98% (Côté-Allard et al., 2019) using deep learning algorithms. In this case, only one Myo armband was used and the sEMG signal was split into 260 ms segments, observing that the classifier accuracy grew with the training repetition. In another work, the accuracy of the classifier was between 81.9 and 90.1% (Wei et al., 2019), the better score was obtained with the multi-view convolutional neural network using both Myo armbands (16 channels) and a maximum of 83.9% using only one Myo armband. Accordingly, to these previous studies, the channel number influences the classifier accuracy; more information presents better results. Also, the deep learning algorithms present good results, but the necessary time to train these algorithms was not presented.

To better understand all processes during neural network training, a dataset with 56 features × 600 samples was used for statistical analysis. Performance of the proposed neural network (output vector of each gesture) was compared statistically using a one-way analysis of variance (ANOVA), in Matlab 2020b. Results were declared statistically significant if they were associated with *p* < 0.05.

The precision, recall, and f-score of the LRN confusion matrix presented in Figure 9 were calculated obtaining a score of 0.99 for precision, 1 for recall, and an F1 score of 0.994.

The proposed classifier presents 98.7% accuracy, but this value can be influenced by the user characteristics and also by the hardware and the method with which the signals were stored. Combining different neural networks presents promising results for gesture recognition, with a reasonable time to acquire new data and retrain neural network architectures. According to the results obtained with the NinaPro dataset, the deep learning architecture presents 25.4% better results (a better result with similar hardware of 83.9%). In our case, the proposed architecture can be easily retrained with a personalized user database (100 samples /gesture), where this number of samples can be a limitation for the deep learning architectures. Also, future works will include a test with different users where the sEMG signal can be altered and where personalized classifiers can be a good approach for a good classification.

A limitation of the proposed method is constraining the user to acquire the data and retrain the algorithm at the beginning of therapy for good results.

## 5. Conclusions

Neural networks are a promising alternative to identify hand gestures depending on the sEMG signals. The proposed architecture - composed by the probabilistic, Bayesian, and temporal networks - offers a good accuracy for hand gesture identification, with a hit rate precision of 98.7%, higher than the previous results mentioned in the literature. Nevertheless, state-of-the-art papers use different gestures which use different muscle groups, so comparison between results is not representative.

This work studied different architectures of shallow and deep neural networks and the best result was reached when combining a probabilistic network in parallel with a Bayesian network and whose results fed a temporal network. To automatically train these architectures, an application was developed which enables sEMG data acquisition for the Myo Armband for a specific user and trains the network's architecture in less than 5 min, guaranteeing a personalized good classification of gestures in real time. Indeed, the application allows the connection with a hand rehabilitation device. Opposite from other solutions, the interface proposed accurately achieved the hand gesture identification (six gestures created only by finger movement), generating reference for the rehabilitation device and a direct connection with it.

According to the hand gesture recognition, a high-level algorithm was developed to generate the necessary reference for the rehabilitation device of the actuators (thumb flexion, thumb opposition, index, middle, ring, and pinky fingers). This algorithm generates the reference according to the user's intention of movement, motivating them to participate in the rehabilitation therapy. In this way, rehabilitation therapy is more effective.

In the future, the rehabilitation device presented in this paper may be improved by adding the extension actuators and connecting the movement of the actuators with each finger position. The proposed algorithm was tested only on a few healthy subjects and more tests need to be applied. Also, the algorithm needs to be tested and validated by different subjects with neural and muscle disorders.

## Data Availability Statement

The original contributions presented in the study are included in the article/Supplementary Material, further inquiries can be directed to the corresponding author/s.

## Ethics Statement

The studies involving human participants were reviewed and approved by the Research Ethics Committee of the Universidad Rey Juan Carlos Madrid, with the internal register number 26/12. Written informed consent to participate in this study was provided by the participants.

## Author Contributions

DB and LM oversaw project administration and funding acquisition. MG-T developed the neural network architectures and carried out the experiments. DC collaborated on experiments and supervised the research. JA developed the application and the high-level algorithm for the rehabilitation device. DC, MG-T, and DB wrote the manuscript. All authors read and approved the final manuscript.

## Funding

For this research, we received funding from the Sistema robótico para propiciar la marcha en niños pequeños con Parálisis Cerebral under Grant PID2019-105110RB-C32/ AEI/10.13039/501100011033, Spanish research project; from RoboCity2030-DIH-CM, Madrid Robotics Digital Innovation Hub, S2018/NMT-4331, funded by Programas de Actividades I&D en la Comunidad de Madrid; and co-funding from Structural Funds of the EU.

## Conflict of Interest

The authors declare that the research was conducted in the absence of any commercial or financial relationships that could be construed as a potential conflict of interest.

## Publisher's Note

All claims expressed in this article are solely those of the authors and do not necessarily represent those of their affiliated organizations, or those of the publisher, the editors and the reviewers. Any product that may be evaluated in this article, or claim that may be made by its manufacturer, is not guaranteed or endorsed by the publisher.
